# Hypoglycaemic activity of biosynthesized copper oxide nanoparticles in alloxan‐induced diabetic Wister rats

**DOI:** 10.1002/edm2.423

**Published:** 2023-04-10

**Authors:** Maimuna Bello Umar, Augustine Innalegwu Daniel, Jimoh Oladejo Tijani, Rebecca Olufemi Akinleye, Enriquay Smith, Marshall Keyster, Ashwil Klein

**Affiliations:** ^1^ Department of Biochemistry Federal University of Technology Minna Nigeria; ^2^ Department of Biotechnology University of the Western Cape Bellville South Africa; ^3^ Department of Chemistry Federal University of Technology Minna Nigeria

**Keywords:** Alloxan monohydrate, biosynthesis, copper oxide nanoparticles, diabetes mellitus, Histoarchitecture

## Abstract

**Background:**

Diabetes mellitus (DM) is a metabolic disorder that affects the body's ability to produce or use insulin. This study evaluated the hypoglycaemic activity of biosynthesized copper oxide nanoparticles (CuO‐NPs) in alloxan‐induced diabetic Wister rats.

**Methods:**

CuO‐NPs were synthesized via the green route and characterized using different analytical tools. Diabetes was induced intraperitoneally using 90 mg/kg body weight of alloxan monohydrate in albino rats. Thirty (30) rats were randomly divided into 5 groups of 6 rats each and orally treated for 21 days. Groups I and II were treated with 300 mg/kg bwt *Cereus hildmannianus* extract and CuO‐NPs, respectively. Groups III and IV received 5 mg/kg bwt of Glibenclamide and 2 mL of normal saline, respectively, while Group V was left untreated as the diabetic control. Blood glucose (BG) levels and body weight changes were monitored at 3‐ and 7‐day intervals, respectively, throughout 21‐day treatment period. Lipid profiles, enzyme assays and histopathological studies of the liver were also carried out.

**Results:**

Spheroidal tenorite phase of CuO‐NPs with a crystallite size of 62.57 nm, surface area (20.64 m^2^/g) and a UV‐maximum absorption at 214.27 nm was formed. The diabetic rats treated with 300 mg/kg bwt CuO‐NPs had the highest BG lowering ability (from 482.75 ± 27.70 to 124.50 ± 2.50 mg/dL). A significant difference (*p* < 0.05) in weight gain and serum enzymes was also observed in the CuO‐NPs treated group compared with other groups. The CuO‐NPs‐treated group had a significant increase (*p* < 0.05) in HDL‐cholesterol and a decrease in total cholesterol, triglycerides, LDL‐cholesterol and VLDL‐cholesterol compared with other groups.

**Conclusion:**

The green synthesized CuO‐NPs nanoparticles significantly reduced (*p* < 0.05) blood glucose levels in rats and other associated indices and could serve as drug lead in the treatment of diabetes.

## Graphical abstract

Hypoglycaemic activity of CuO‐NPs was carried out in this study. The study shows the potential of CuO‐NPs in reducing blood sugar level in albino Wister rats.

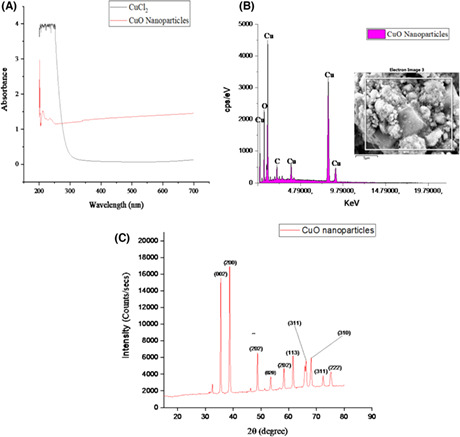

## 1 INTRODUCTION

Diabetes mellitus (DM) is a chronic metabolic disorder caused by an alteration in insulin secretion, insulin action or both.^1^ It is characterized by persistent hyperglycaemia and problems in carbohydrate, lipid and protein metabolism. Long‐term damage is one of the outcomes of DM with diverse organ malfunction and failure.^1^, ^2^ There are an estimated 143 million persons living with diabetes globally and by 2030; this figure is expected to double.^3^, ^4^, ^5^ There are three forms of DM.^6^, ^7^ Type 1 diabetes (insulin‐dependent DM) is an autoimmune disease that occurs when the β‐cells of the pancreas are damaged, resulting in the pancreas producing little or no insulin. It most commonly affects children and young adults.^6^ Type 2 diabetes, commonly known as ‘insulin‐dependent DM’, is the most common type of diabetes in adults, accounting for more than 90% of cases. Insulin resistance is a condition in which the pancreas generates enough insulin but the body is unable to use the insulin efficiently.^3^, ^7^ The third form of diabetes is gestational DM (GDM), which is a type of glucose intolerance that develops or is first noticed during the second or third trimester of pregnancy. One of the most common metabolic disorders during pregnancy is GDM. It is caused by a lack of insulin, or a hormone produced during pregnancy.^6^, ^7^ Although a wide range of synthetic drugs have lately been used to treat T2DM, the majority of them have significant long‐term side effects, including drug resistance, hepatotoxicity, abdominal pain, flatulence and diarrhoea.^3^, ^4^ As a result, a search for an alternate drug with hypoglycaemic effects on T2DM is required.

In the last few decades, advances in nanotechnology have played a key role in the medical, pharmaceutical, agricultural and textile industries.^8^ Metallic nanoparticles such as silver, zinc and gold have been reported to have therapeutic potentials.^9^, ^10^ Oxides of transition metals such as ZnO, CuO, TiO_2_, Fe_3_O_4_ and NiO nanoparticles have found application as advanced nano‐substances in various fields of life such as energy, environmental and biomedical field due to their high surface area and adsorption capacity.^8^ Different researchers have reported that metallic oxide nanoparticles have better biological activities than metallic nanoparticles.^11^, ^12^, ^13^, ^14^, ^15^

Copper is one of the most common transition metals found in metabolic pathways. Several transition metals have catalytic activity and oxygen transport potentials into the host active sites as cofactors.^3^ For instance, the biocompatibility of CuO‐NPs reduces the danger of toxicity, which is a key issue for other metallic nanoparticles with medical applications. Hence, biologically synthesized metallic oxide nanoparticles with a wide range of therapeutic potentials have recently gained attention from researchers.^16^, ^17^, ^18^, ^19^, ^20^ CuO‐NPs have also been reported to have anticancer, antimicrobial and antioxidant properties thus, suitable as a viable tool for biomedical applications.^21^, ^22^

CuO‐NPs have been synthesized via different routes using physical and chemical approaches such as microwave irradiation, thermal decomposition, sol–gel, colloidal thermal synthesis, sonochemical, hydrothermal and quick precipitation.^23^ However, these methods are labour and energy intensive, expensive and involve the release of hazardous chemicals into the environment.^24^, ^25^ Currently, there is a shift from the physical and chemical methods of synthesis of nanoparticles to the biological method known as biosynthesis or green synthesis.^15^, ^26^ The phytosynthesis of CuO‐NPs has gained more attention recently owing to its sustainability, cost‐effectiveness and simplicity.^8^ Green synthesis involves the use of bacteria, fungi, algae, yeast and plant extracts or their products as reducing agents for the synthesis of metallic or metallic oxide nanoparticles, which support biocompatibility and large‐scale production.^27^ Several authors have reported the green synthesis of CuO‐NPs using different plant extracts^27^, ^28^, ^29^, ^30^, ^31^, ^32^ including their antibacterial potentials.^33^, ^34^ In addition, Ghosh et al.^3^ reported on the in vitro antidiabetic activity of CuO‐NPs synthesized using *Dioscorea bulbifera* extract and found out that the nanoparticles significantly inhibited α‐amylase and α‐glucosidase, which are considered to be significant pharmacological targets for the treatment of type 2 DM. Different researchers have also investigated the in vitro antidiabetic activity of CuO‐NPs [2]. This is the first comprehensive report on the in vivo hypoglycaemic activity of biosynthesized CuO‐NPs in alloxan‐induced Wister rats. This study evaluated the hypoglycaemic activities of biosynthesized CuO‐NPs in alloxan‐induced Wister rats. The green synthesis of CuO‐NPs was carried out using copper (II) chloride dihydrate and an extract of *Cereus hidmannianus* as the precursor. The prepared CuO‐NPs were then characterized for their morphology and microstructure, elemental composition, absorption bands and surface area. The in vivo activity of the biosynthesized CuO‐NPs in Wister rats was also examined. Finally, lipid profiles, enzyme assays and histopathological studies of the liver were also carried out.

## 2 MATERIALS AND METHODS

### 2.1 Materials

Analytical grade copper (II) chloride dihydrate (CuCl_2_.2H_2_O, 99.9%) and sodium hydroxide pellets (NaOH, 99%) were purchased from Merck, India. Alloxan monohydrate (Sigma Aldrich, Bangalore, India), Glibenclamide (Daonil; Aventis Pharma. Ltd., India) was purchased from BDH. All chemicals are used without further purification.

### 2.2 Collection and processing of plant materials

The fresh stem of *C. hildmannianus* was collected from the environment of the Federal University of Technology, Minna, Niger State, Nigeria. The stems were washed with clean water, cut into smaller pieces and then air‐dried at room temperature at the Biochemistry Department Laboratory of the Federal University of Technology Minna for 3 weeks. The dried stems were pulverized and blended into a fine powder using mortar with a pestle and a kitchen‐type blender.

Five hundred grams (500 g) of the powdered sample was extracted exhaustively with methanol under reflux at 40°C for 2 h. The extract was filtered using a muslin cloth and Whatman No 1 filter paper. The extract was left open in a fume hood for the methanol to evaporate, and a semisolid brown‐like paste was finally obtained as the extract. The extract was preserved in a refrigerator at 4°C until required for use.

### 2.3 Quantitative phytochemical analysis of *C. hildmannianus* extract

The total flavonoids, total phenols and tannins contents of the plant extract were determined using the modified method reported by Daniel et al.^1^

### 2.4 Green Synthesis of Copper Oxide nanoparticles

Twenty gram (20 g) of the powdered plant was weighed and put into a 500 mL beaker containing 400 mL of distilled water. The mixture was stirred using a magnetic stirrer for about 30 min at 40°C. The brownish mixture was filtered using filter paper to obtain a fine filtrate. Copper chloride dihydrate (5.294 g) was weighed and dissolved in 100 mL of distilled water to obtain a concentration of 0.3 M followed by heating on a magnetic stirrer at 150 rpm for about 30 min until the solution changes from blue to green coloration. Exactly, 20 mL of the extract was added under continuous stirring while 20 mL of 0.5 M NaOH solution was added dropwise to raise the pH value to 9 and stirred further for 30 min. This was accompanied by the formation of precipitates, which were left to age overnight and then filtered using a Whatman No 1 filter paper. The precipitates were separated from the aqueous extract, first by decantation, and then washed severally with distilled water until the filtrate was clear. The washed precipitates obtained were dried in an oven at 80°C for 3 h.

### 2.5 Characterization of Copper oxide Nanoparticles

The absorption band of the synthesized CuO‐NPs was characterized using a double‐beam UV–visible spectrophotometer (Shimadzu UV‐1800) at a wavelength range of 200–800 nm, energy‐dispersive X‐ray spectroscopy (EDX) (Zeiss Aunga), high‐resolution transmission electron microscopy (HRTEM) (Zeiss Aunga), high‐resolution scanning electron microscope (HRSEM) (Zeiss Aunga), X‐ray diffraction (XRD) (Bruker d8), Brunauer–Emmett–Teller (N_2_ BET) using NOVA 4200e surface area and pore analyser instrument and X‐ray photoelectron spectroscopy (PHI 5400) couple with a hemispherical sector analyser to confirm the physicochemical properties of the synthesized CuO‐NPs.

### 2.6 Experimental animals/ethics

Thirty (30) Wistar rats of either sex aged 8–12 weeks old weighing between 100 and 150 g were purchased from the animal farm of the University of Jos, Jos, Plateau State, Nigeria. The rats were kept in well‐ventilated metal cages and maintained at room temperature of 28 ± 2°C, 45–55% of relative humidity on a 12‐h light/12‐h dark cycle, with access to water and pelletized commercial livestock feed (grower) ad libitum. The rats were kept for 2 weeks to acclimatize to the environmental conditions.

### 2.7 Induction of diabetes

Diabetes mellitus was induced in the rats by a single intraperitoneal (i.p.) injection of 90 mg/kg bwt of alloxan monohydrate (Sigma, St. Louis, USA) in PBS (pH = 7.4).^35^ Animals with fasting plasma glucose concentration (FPGC) > 111 mg/dL, measured using Fine test Auto‐coding Premium Blood Glucose Monitoring System for self‐testing, for 5 consecutive days were considered diabetic and selected for the study. A total of thirty (30) Wistar rats of either sex were divided into 5 groups of 6 rats each. The animals were deprived of food and water for an additional 16 h before commencement of treatment.^35^ All procedures were conducted in accordance with the guidelines for the care and use of laboratory animals (USA National Institute of Health Publication No 80‐23, revised 1996) and were reviewed and approved by the Animal Ethics Committee of the Federal University of Technology Minna, Nigeria, on the 8th of February 2022 and assigned a number: 000021.

### 2.8 Experimental design

The rat groups were assigned on the basis of the treatments received by oral gavage on a daily basis for 21 days as follows: Group I: Diabetic rats treated with 300 mg/kg bwt of methanol extract of *C. hildmannianus*, Group II: Diabetic rats treated with 300 mg/kg bwt of CuO‐NPs, Group III: Diabetic rats received 5 mg/kg bwt of Glibenclamide (standard drug), Group IV: Normal control rats received 1.0 mL/kg bwt of PBS, Group V: Diabetic control rats. The body weight of rats in each group was also determined at every 7‐day intervals.

### 2.9 Collection and preparation of blood and tissues

On the 21st day of the experiment, overnight fasted rats were euthanized using 150 mg/kg bwt of sodium pentobarbitone anaesthesia; afterwards, blood samples were collected via cardiac puncture from each mouse into a plain sample bottle. The blood samples were kept at room temperature for 2 h to coagulate and then centrifuged at 3000x ***g*** for 10 min; then serum was separated with clean Pasteur pipette and store frozen until used for biochemical analysis. The liver of rats was excised, rinsed in normal saline and preserved in 10% formol saline (v/v) for histopathological study.^36^

### 2.10 Biochemical assays

The biochemical parameters of the serum samples of the treated and untreated rats at the end of 21‐day treatment were assayed using appropriate kits. High‐density lipoprotein (HDL), alanine aminotransferase (ALT), alkaline phosphatase (ALP) and aspartate aminotransferase (AST), Total cholesterol (TC) and triglyceride concentrations (TRIG) were determined as described by Oluba et al.,^37^ Additionally, serum LDL‐C concentration was determined according to the formula described by Friedewald et al.^38^ and reported by Oluba et al.^37^

(1) $$\left[\mathrm{LDL}-C\right]=\left[\mathrm{TC}\right]-\left[\mathrm{HDL}-C\right]-\frac{\mathrm{TAG}}{5}$$

Serum VLDL‐C concentration was estimated using the methods of Burnstein and Sammaille,^39^ where the ratio of serum VLDL‐C to triglyceride concentrations was fixed at 1:5 in fasting animals.

(2) $$\left[\text{VLDL}-C\right]=\frac{\mathrm{TAG}}{5}$$

### 2.11 Statistical and data analyses

The data collected were presented as mean ± SEM and analysed using a one‐way analysis of variance (ANOVA), while treatment means were separated by the least significant difference (LSD) incorporated in the IBM statistical package for social sciences (SPSS) version 20 (IBM Corp., Armonk, N.Y., USA).

## 3 RESULTS

### 3.1 Quantitative phytochemicals constituents of methanol stem extract of *C. hildmannianus*

The crude extract of *C. hildmannianus* leaf showed the presence of phenols, flavonoids and tannins as shown in Table 1 below. The result shows that phenol has the highest concentration (86.1 ± 0.21 mg/g) with flavonoids having the least concentration (1.61 ± 0.15 mg/g).

**TABLE 1:** Phytochemical constituent of methanol stem extract of *C. hildmannianus*.

Phytochemicals	Amount (mg/g)
Phenols	86.12 ± 0.21
Flavonoids	1.61 ± 0.15
Tannins	56.35 ± 0.13

*Note*: Values are presented as mean ± standard error of the mean (SEM) of three replicates.

### 3.2 Characterization of copper oxide nanoparticles

**FIGURE 1:**
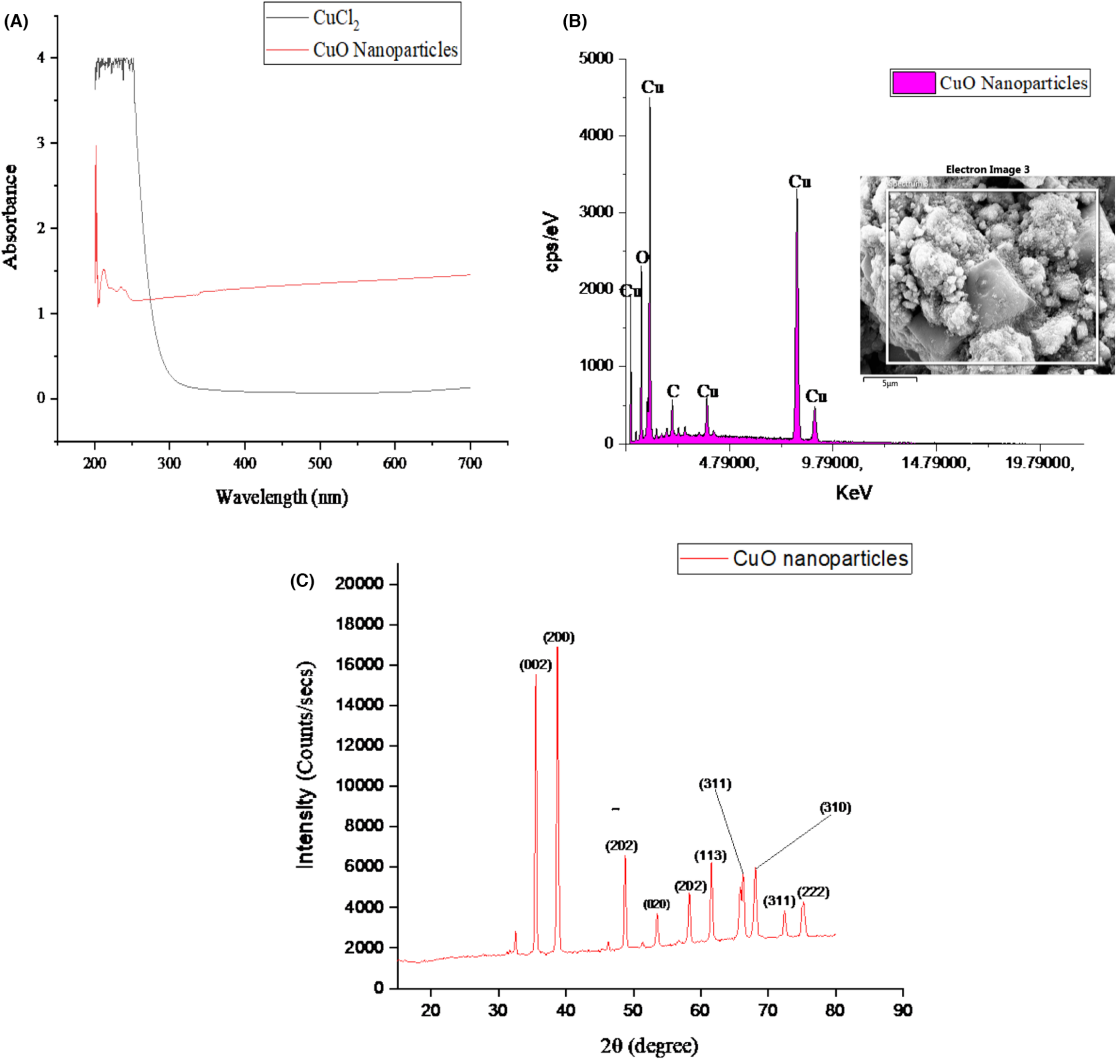
(A) UV–vis spectrum, (B) elemental composition and (C) X‐ray diffraction pattern of the green synthesized CuO‐NPs using stem extract of *C. hildmannianus*.

Characterization of the synthesized CuO‐NPs was carried out using a UV–visible spectrophotometer, energy‐dispersive X‐ray spectroscopy (EDX) and X‐ray diffraction (XRD) pattern as shown in Figure 1A–C, respectively. The nanoparticles have a maximum UV absorption of 214.27 nm, and the elemental composition shows the presence of copper (Cu), oxygen (O) and carbon (C) as the most abundant element present. The XRD analysis shows a spheroidal tenorite structure of the nanoparticles at different peak positions.

**FIGURE 2:**
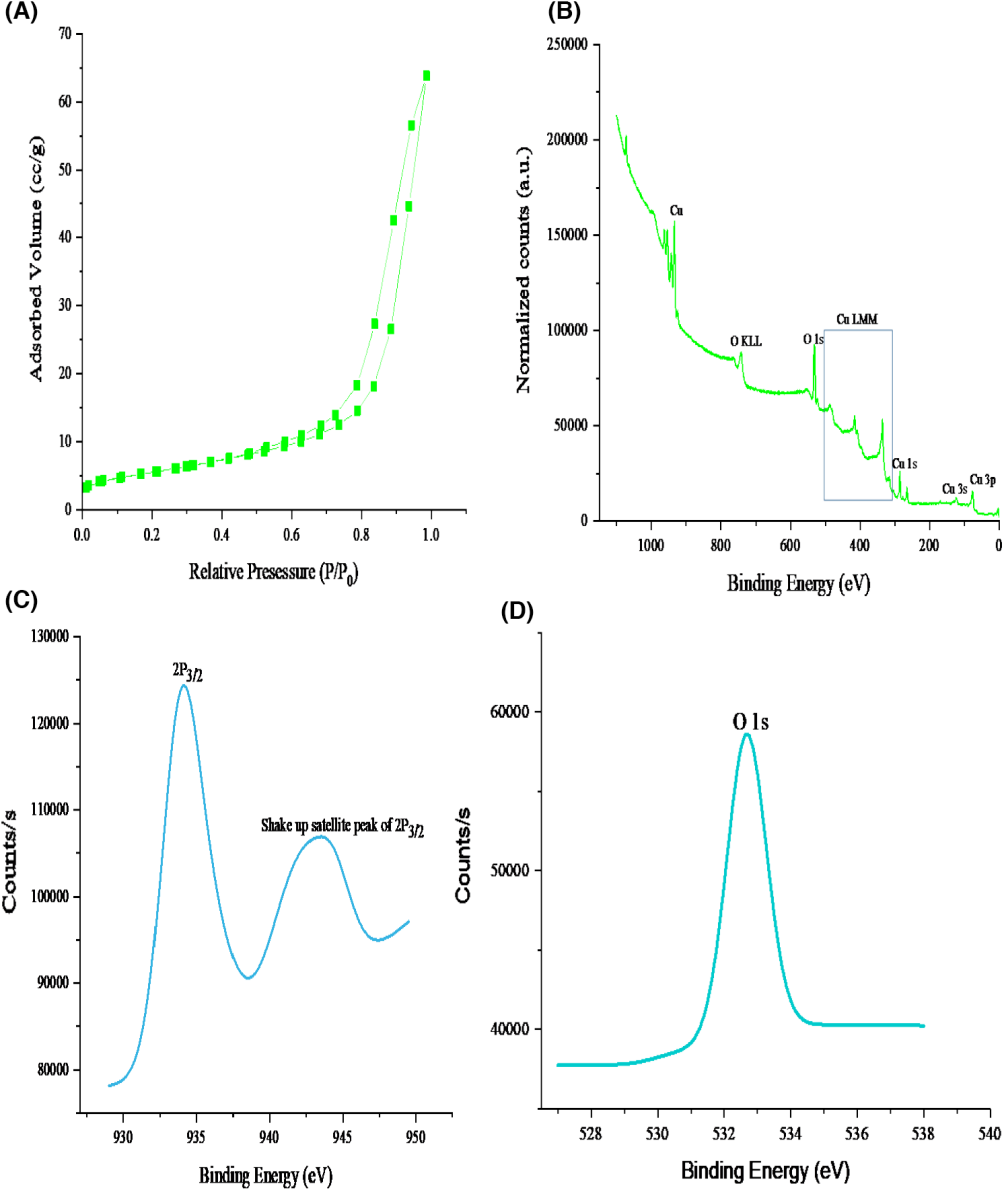
(A) BET nitrogen adsorption–desorption isotherms of CuO‐NPs (B) XPS survey of CuO‐NPs (C) XPS spectrum of Cu 2p and (D) O 1s of CuO‐NPs.

The BET plot of nitrogen adsorption–desorption isotherms of CuO‐NPs is presented in Figure 2. The isotherm plot of the CuO‐NPs according to IUPAC classification belongs to type IV with a narrow hysteresis which represent the mesoporous nature of CuO‐NPs.^40^ The nanoparticle has a large surface area of 20.640 m^2^/g, a pore diameter of 11.517 nm and a pore volume of 0.08 cc/g (Figure 2A). The surface oxidation state of the elements in CuO‐NPs was examined using XPS containing Cu and O at different binding energies belonging to 1s, 3s and 3p orbitals (Figure 2B). The XPS spectrum of Cu 2p and O 1s of CuO‐NPs (Figure 2C,D) shows that the spectrum was calibrated with a binding energy of 285.65 eV for C 1s electron.

**FIGURE 3:**
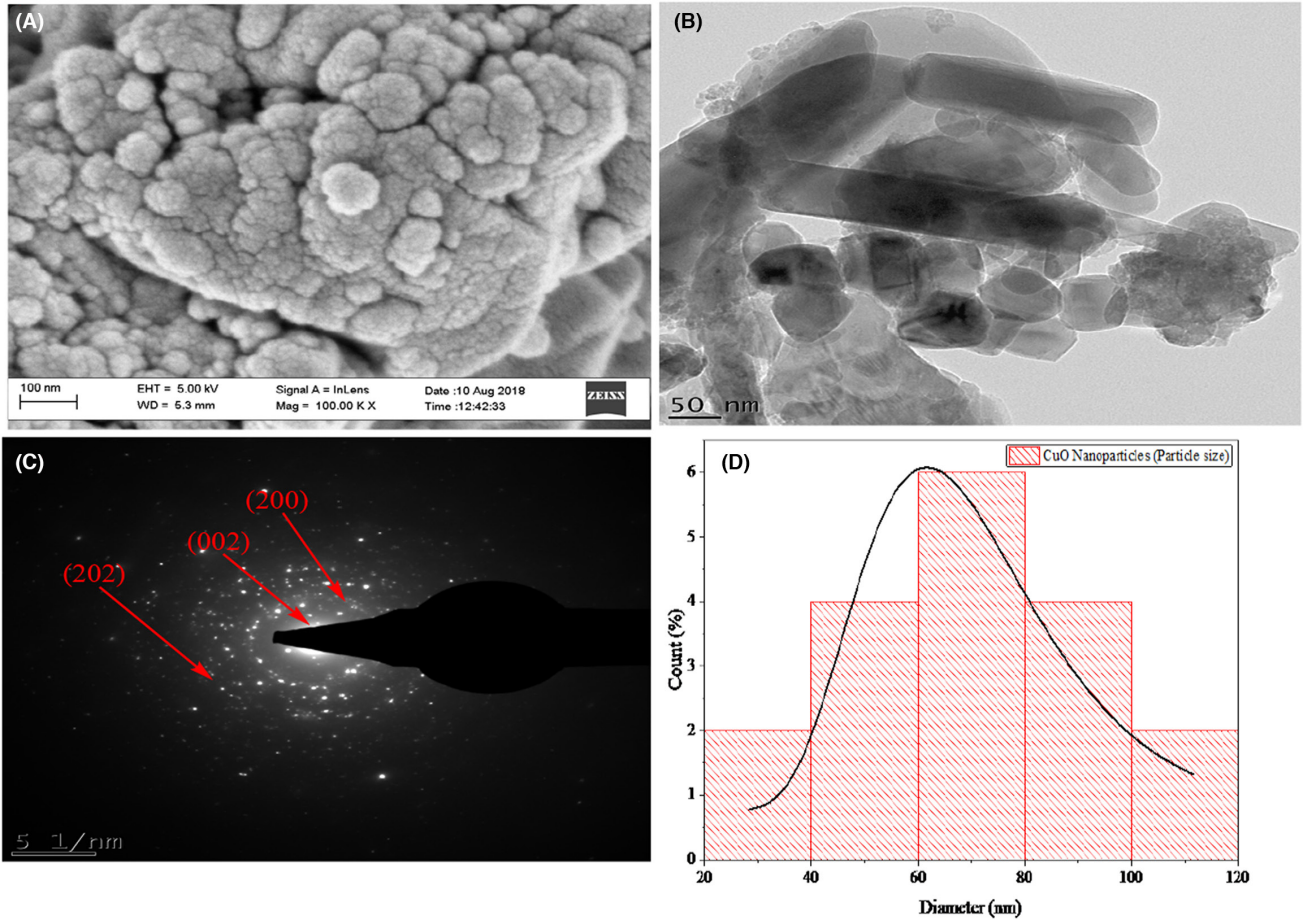
(A) High‐resolution scanning electron microscopy image, (B) High‐resolution transmission electron microscopy image, (C) selected area electron diffraction patterns and (D) particle size distribution of green synthesized CuO‐NPs using stem extract of *C. hildmannianus*.

High‐resolution scanning electron microscope (HRSEM) was further used to determine the morphology of the synthesized nanoparticles, which indicated that the synthesized nanoparticles are agglomerated spherical particles of different sizes (Figure 3A). The high aggregation/agglomeration of the nanoparticles may be attributed to the polarity and electrostatic force of attraction between the copper salt precursor and plant extract used.^41^ High‐resolution transmission electron microscope (HRTEM) of the nanoparticles revealed the presence of spherical and rod‐shaped nanoparticles (Figure 3B). The HRTEM image and the selected area electron diffraction (SAED) pattern (Figure 3C) acquired from an area containing many monodispersed nanoparticles which corroborated with the XRD data. The nanoparticles average particle size ranges between 60 nm (Figure 3D).

### 3.3 Effect of green synthesized CuO‐NPs on fasting blood glucose levels, body weight changes, lipid profiles and serum enzyme levels of alloxan‐induced diabetic Rats

**FIGURE 4:**
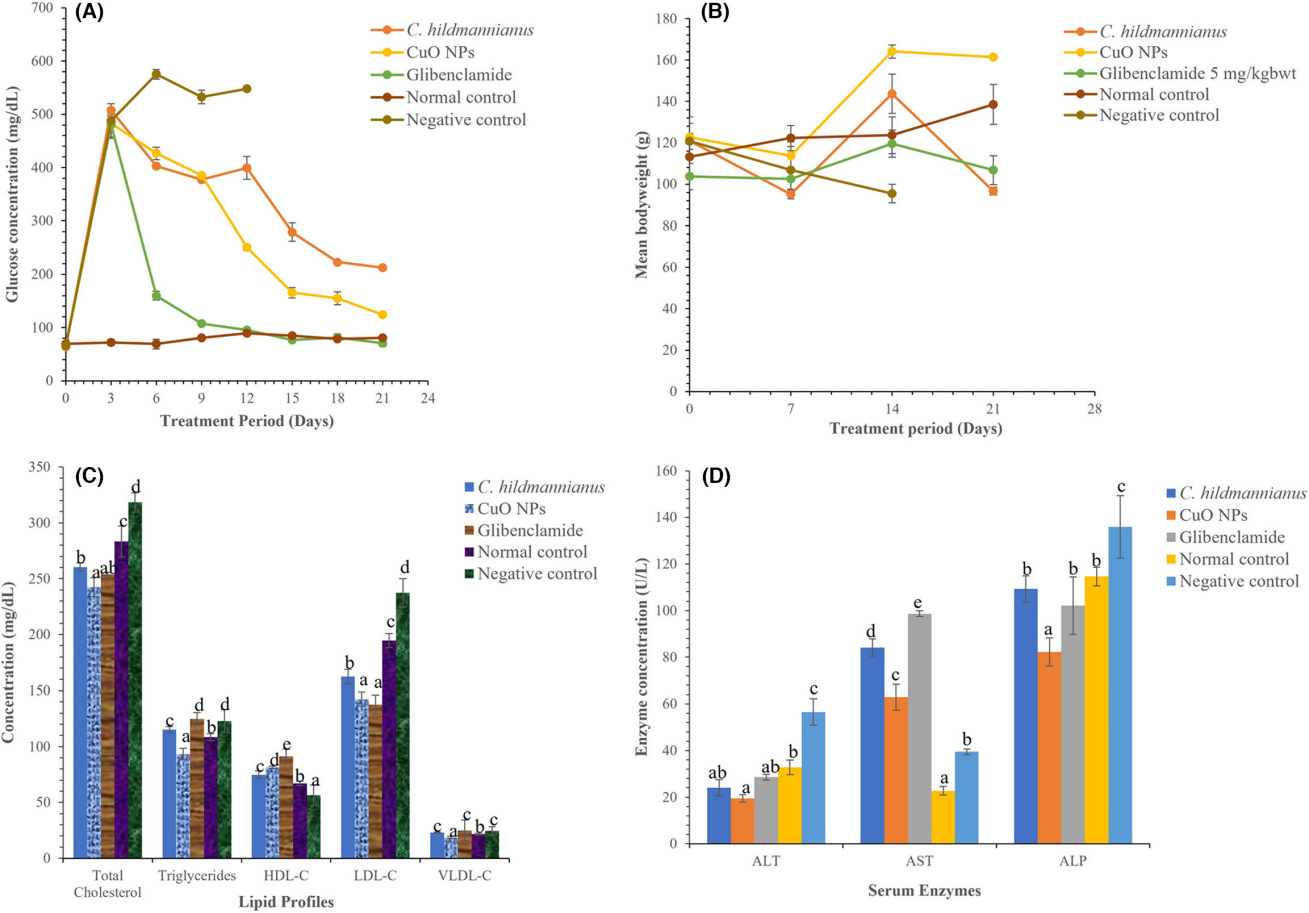
(A) In vivo antidiabetic activity of green synthesized CuO‐NPs on fasting blood glucose levels, (B) body weight changes, (C) lipid profiles and (D) serum enzyme levels of alloxan‐induced diabetic rats. Bars of the same group with different alphabet are significantly at *p* < 0.05.

Figure 4A shows the in vivo antidiabetic activity of CuO‐NP and the aqueous extract of *C. hildmannianus*. The nanoparticles significantly lowered (*p* < 0.05) blood glucose levels to below 200 mg/dL after 21 days of treatment which is comparable with the standard antidiabetic drug (Glibenclamide). The extract also shows the ability to clear the blood glucose levels but could not go below 200 mg/dL, while the negative control group could not survive beyond Day 12 due to the continued rise in their blood glucose levels. The effect of CuO‐NPs on the body weight of alloxan‐induced diabetic rats is presented in Figure 4B. The group treated with CuO‐NPs show a significant gain (*p* < 0.05) in their body weight compared with those treated with extract of *C. hildmannianus* and Glibenclamide. The lipid profile of rats treated with CuO‐NPs in Figure 4C shows a significant decrease (*p* < 0.05) in plasma cholesterol, triglycerides, low‐density lipoprotein (LDL) and very‐low‐density lipoproteins (VLDL) with an increase in high‐density lipoproteins (HDL). There was a significant decrease (*p* < 0.05) in the serum ALT, AST and ALP of rats treated with CuO‐NPs compared with the extract control and Glibenclamide groups (Figure 4D).

### 3.4 Effect of copper oxide nanoparticles on the liver histoarchitecture of the rats

**FIGURE 5:**
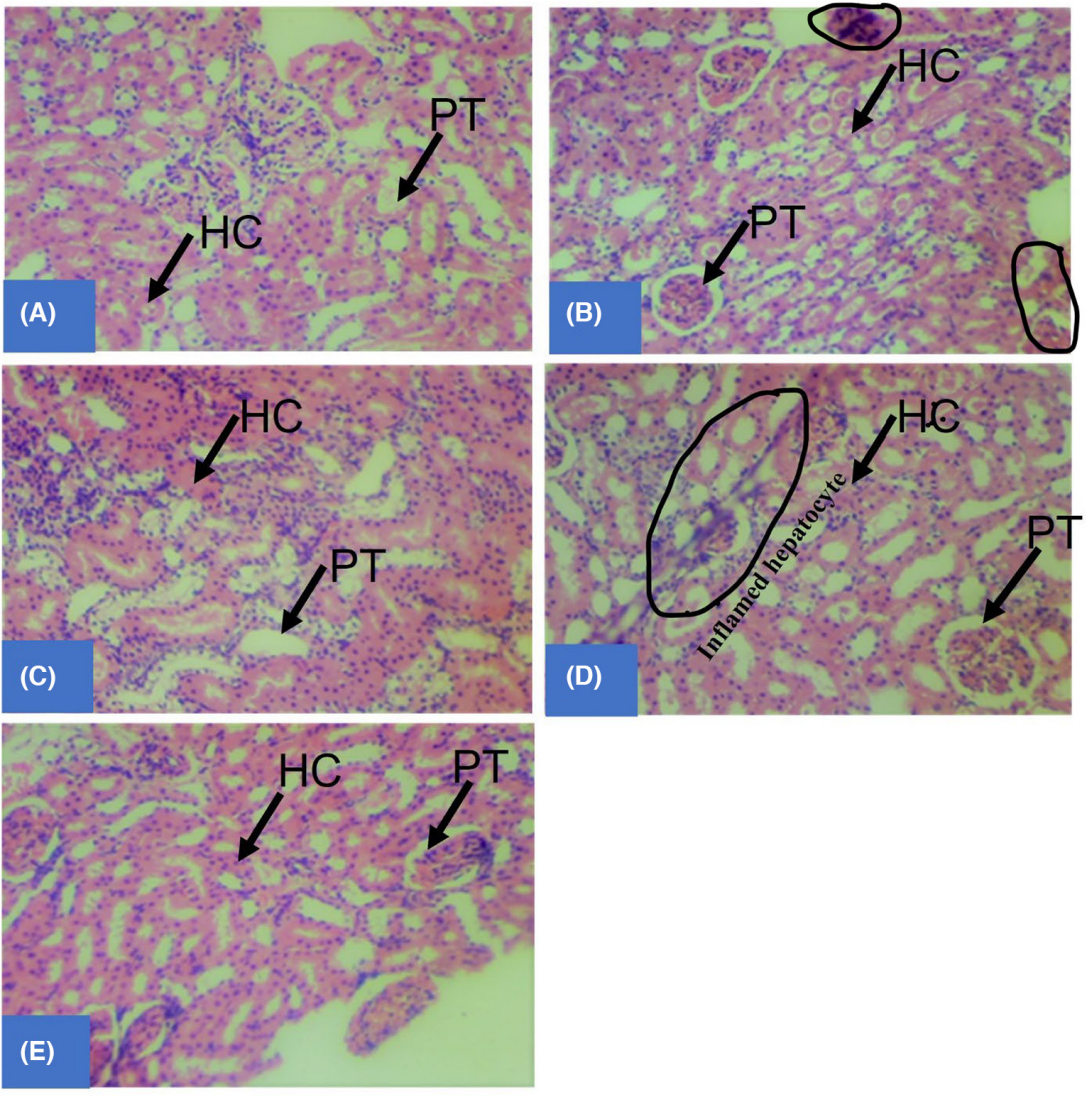
Histoarchitecture of liver sections of diabetic rats treated with 300 mg/kg bwt of *C. hildmannianus* extract (A), 300 mg/kg bwt of CuO nanoparticles (B), 5 mg/kg bwt of Glibenclamide (C), diabetic control (D) and normal saline (E), respectively, showing hepatic tissue with preserved architecture composed of cords of normal hepatocytes, normal portal tracts and central vein. There are no features of acute or chronic damage in the organs of the experimental rats. Keys: HC, Hepatic cells; PT, Portal tract.

Histopathological examination of the liver of rats treated with 300 mg/kg bwt of *C. hildmannianus* and CuO‐NPs shows that the extract and CuO‐NPs had no adverse effects on the histoarchitecture of the organs (Figure 5) while rats treated with CuO‐NPs show hepatic haemorrhage in the liver.

## 4 DISCUSSION

The synthesis of CuO‐NPs was successfully carried out via the green methods using an extract of *C. hildmannianus*. The synthesis of metallic oxide nanoparticles requires three major components, which are reducing agents, stabilizing agents and solvent medium.^42^, ^43^, ^44^ Although different metallic nanoparticles have been synthesized using biological materials such as plants, bacteria, fungi and algae, the actual mechanism of synthesis remains unknown with novel methods evolving each day hence, presenting opportunities for further study.^45^ When nanoparticles are synthesized using plant extract, all the phytochemicals contained in the extract are utilized in the process without targeting a specific phytochemical. These phytochemicals play a major role as reducing and capping agents in a variety of ways. Different classes of phenols such as caffeic and gallic acid have been reported to act as reducing agents in the synthesis of metallic oxide nanoparticles.^45^ The formation of a transitional complex between Cu^2+^ ion and phenolic hydroxyl groups in plant extract and via oxidation process, which changes to quinine resulted in the formation of CuO‐NPs. Studies have shown that polyols such as flavones and terpenoids with polysaccharides contents in plant extract act synergistically in metal ion reduction.^45^ Hence, the presence of these phytochemicals in the extract of *C. hildmannianus* plays a major role in the synthesis of CuO‐NPs.

The UV–vis spectrum of CuO nanoparticles synthesized using the stem extract of *C. hildmannianus* with a maximum absorption spectrum of 214.27 nm (Figure 1A) can be attributed to the formation of cuprous oxide nanoparticles. A similar peak (220 nm) was also reported for CuO‐NPs by Renuga et al.^46^ UV–vis spectroscopy analysis is the first physical visible step for confirming the synthesis of nanoparticles through colour changes. In the synthesis of CuO‐NPs using stem extract of *C. hildmannianus*, there was a colour change from the pale blue of the copper solution to green upon stirring for 30 min and finally to dark green after the addition of the plant extract and subsequent adjustment of pH using NaOH as the base. The colour change may be due to the reaction between copper (II) chloride dihydrate and sodium hydroxide to form copper (II) hydroxide (1), which further reacts with the phytochemical contents such as phenols and flavonoids of the plant to form dehydroascorbic acid and copper (I) oxide (2).

(1) $${\text{CuCl}}_{2}.{\text{2H}}_{2}O+\text{2NaOH}\to \mathrm{Cu}{\left(\mathrm{OH}\right)}_{2}+\text{2NaCl}+{\text{2H}}_{2}O$$

(2) $$\text{2Cu}{\left(\mathrm{OH}\right)}_{2}+C_{6}H_{8}O_{6}\to {\mathrm{Cu}}_{2}O+C_{6}H_{6}O_{6}+{\text{3H}}_{2}O$$

With further stirring and oven drying at 80°C for 3 h, copper (I) oxide was converted to CuO‐NPs via a series of reaction steps (3–5).^47^

(3) $${\mathrm{Cu}}_{2}O+{\mathrm{OH}}^{-}+H_{2}O\;{\left[\mathrm{Cu}{\left(\mathrm{OH}\right)}_{2}\right]}^{-}+\text{CuOH}$$

(4) $$\text{2CuOH}\to {\mathrm{Cu}}_{2}O+H_{2}O$$

(5) $$\text{4CuOH}+O_{2}\to \text{4CuO}+{\text{2H}}_{2}O$$

CuO‐NPs were synthesized as spherical‐shaped nanoparticles after the reduction of CuO material by the action of plant phytochemicals.^48^ Further confirmation of the biosynthesized CuO‐NPs using energy‐dispersive X‐ray spectroscopy (EDX) shows that the nanoparticles consist majorly of Cu and O with a trace of C with an atomic weight of 55.72%, 34.57% and 9.70%, respectively (Figure 1B). The presence of carbon maybe attributed to the phytochemical constituents of the extract such as phenols, flavonoids and tannins, which are present in the extract.^49^ The crystal formation of the synthesized CuO nanoparticles was determined using X‐ray diffraction (XRD) analysis (Figure 1C). The result revealed the spheroidal tenorite structured with peaks at 35.42°, 38.90°, 48.72°, 53.49°, 61.53°, 66.22°, 66.45°, 72.37° and 75.25° at 2θ values to crystal planes of (002), (200), (202), (020), (113), (311), (310), (311) and (222), respectively, that match well with the JCP2_48‐1548. A similar result was also reported for CuO‐NPs using extract of *Syzygium alternifolium* by Yugandhar et al.^47^ The average particle size of the nanoparticles was calculated using the Debye–Scherrer equation to be 13.05 nm (Figure 3D). The crystallite size reported here is lower than those reported by Andualem et al.^34^ who reported a crystallite size of 18.20, 25.30 and 28.10 nm using the varying ratios of plant extract to the copper solution.

Figure 2A shows the BET plot of nitrogen adsorption–desorption isotherms of CuO‐NPs. The isotherm plot of the CuO‐NPs according to IUPAC classification belongs to type IV with a narrow hysteresis, which represents the mesoporous nature of CuO‐NPs.^40^ The nanoparticle has a large surface area of 20.640 m^2^/g, a pore diameter of 11.517 nm and a pore volume of 0.08 cc/g. The result of this study is at variance with the report of Manasa et al.,^40^ and Navada et al.,^50^ who reported a surface area of 17.10 m^2^/g. The variation in the result may be because of different reaction conditions used in the synthesis of the nanoparticles. Small particle size and high surface area play a key role in determining the biological activity of nanoparticles causing an improvement in surface reactivity.^51^, ^52^ The surface oxidation state of the elements in CuO‐NPs was examined using XPS as shown in Figure 2B. The nanoparticle‐containing Cu and O at different binding energies belonging to 1s, 3s and 3p orbitals. The binding energy of 934.10 and 943.56 eV corresponds to Cu 2p_3/2_ and Cu 2p_1/2_, respectively, which is similar to the reported data of Saif et al.^53^ The splitting between these two states is about 9.46 eV, which confirmed the existence of Cu in +2 oxidation state. The binding energy at 532.69 eV corresponds to O 1s of CuO‐NPs. Similar binding energy (529.8 eV) was also reported by Tamuly et al.^54^ and Saif et al.^53^ The XPS analysis strongly confirmed the absence of Cu_2_O and Cu(OH)_2_ as impurities within the sample and proved that CuO nanoparticles consist of only Cu^2+^ and O^2−.^

Blood glucose is the key biomarker used for diagnosis and prognosis of DM. According to Figure 4A, it was noticed that treatment with 300 mg/kg bwt of CuO‐NPs decreased blood glucose levels significantly (*p* < 0.05) when compared to 300 mg/kg bwt of *C. hildmannianus* and the diabetic control (Figure 4A). This agrees with a previous report by Martín Giménez et al.^55^ that CuO‐NPs are good therapeutic agents for the management of type 2 DM. One of the most promising tools in type 2 diabetes management is the use of natural health products. Several trace metals such as chromium, selenium, vanadium, molybdenum and magnesium have been reported to be a good therapeutic agent for lowering blood glucose^33^ because of their insulin‐mimetic actions, they have hypoglycaemic activity.^56^, ^57^, ^58^, ^59^

The ability of alloxan to induce weight loss in untreated rats' mimics what is commonly observed in clinical diabetes. Alloxan induces diabetes by destroying the β‐cells of the islets of Langerhans in the pancreas prompt a decreased degree of endogenous insulin accordingly influencing glucose uptake by tissue.^60^ Glibenclamide, an example of sulfonylurea drugs, fortifies β‐cells to deliver insulin along these lines bringing down the blood glucose level.^61^ A major characteristic of hyperglycaemia is a serious loss of body weight, which might be due to the breakdown of muscle fat and proteins.^60^ In Figure 4B, it was noticed that the body weight of diabetic rats treated with the CuO‐NPs increased significantly (*p* < 0.05) when compared to groups treated with extract and Glibenclamide. This suggests that the nanoparticles were able to reverse the weight loss because of alloxan induction in the animals. The improvement in the body weight seen in hyperglycaemic rats treated with CuO‐NPs might be because of improved metabolic activities making the body more capable of maintaining blood glucose haemostasis.^60^

Diabetes is also associated with an alteration in lipid profiles and other biochemical parameters of patients. In Figure 4C, there was a significant decrease in plasma cholesterol, triglycerides, LDL, and VLDL with an increase in HDL levels for diabetic rats treated with nanoparticles compared with the diabetic control and extract group. On the contrary, a significant increase (*p* < 0.05) in serum lipids level was observed in the alloxan‐induced rats treated with CuO‐NPs when compared to the normal control. This may be a result of a disturbance in the regulation of the activity of the hormone‐sensitive enzyme lipase by insulin due to its deficiency or absence caused by the destruction of the β‐cells of the islet of Langerhans by alloxan.^62^ The decrease in the various lipid profile parameters of the diabetic rats may be due to impaired fatty acids synthesis, enhanced catabolism of VLDL, activation of Lecithin: Cholesterol Acyltransferase (LCAT), tissue lipases, inhibition of acetyl‐CoA carboxylase and production of triglycerides precursors such acetyl‐CoA and glycerol phosphate by CuO‐NPs.^63^ Also, a significant decrease (*p* < 0.05) in the serum ALT, AST and ALP of rats treated with CuO‐NPs compared with the extract control and Glibenclamide groups is observed in Figure 4D. Serum AST, ALT and ALP are enzyme biomarkers used in monitoring liver structural integrity and help in the clinical diagnosis of liver toxicity.^64^ Increased serum liver enzymes are an indication of hepatocellular destruction typical to diabetic complications.^65^ The result, therefore, indicates that the nanoparticles may not be responsible for any injury in the liver of the rats as observed in the histopathological study of the liver of rats treated with the extract and CuO‐NPs (Figure 5). The report of this study is contrary to that of Abdelazeim et al.^62^ who observed an elevation in serum enzymes as a result of an injury in the liver of rats treated with CuO‐NPs. Mohammadyari et al.^66^ also reported that CuO‐NPs increase the ratio of ALT/AST in the serum, which is an indication of cellular leakage. Finally, elevated level of stress has also been reported to be responsible for triggering several ailments, particularly hepatic inflammation.^35^ Therefore, the observed alteration in the serum enzymes of the experimental animals may be due to the stress they undergo because of alloxan induction and the process of treatment for 21 days.

In conclusion, the antidiabetic potentials of CuO‐NPs were successfully evaluated in this study. The nanoparticles significantly lowered (*p* < 0.05) blood glucose levels, plasma cholesterol, triglycerides, LDL and VLDL and an increase in HDL levels of alloxan‐induced diabetic rats after 21 days of treatment. Antidiabetic activities of the CuO‐NPs were linked to its crystalline nature and enhanced surface area for improved absorption and distribution. Rats treated with CuO‐NPs show no alteration of liver architecture or injury. Therefore, it is recommended that further study on the toxicity and safety of this nanoparticle in animal models should be carried out so as to validate the report of this study and justify its usage and safe dose.

## AUTHOR CONTRIBUTIONS

**Maimuna Bello Umar:** Conceptualization (equal); data curation (equal); formal analysis (equal); investigation (equal); methodology (equal); project administration (equal); software (equal); supervision (equal); validation (equal); visualization (equal); writing – original draft (equal); writing – review and editing (equal). **Augustine Innalegwu Daniel:** Conceptualization (lead); data curation (equal); formal analysis (equal); investigation (equal); methodology (equal); project administration (equal); resources (equal); software (equal); supervision (equal); validation (equal); visualization (equal); writing – original draft (equal); writing – review and editing (equal). **Jimoh Oladejo Tijani:** Conceptualization (equal); formal analysis (equal); methodology (equal); supervision (equal); writing – original draft (equal); writing – review and editing (equal). **Rebecca Olufemi Akinleye:** Formal analysis (equal); investigation (equal); methodology (equal); validation (equal); writing – original draft (equal); writing – review and editing (equal). **Enriquay Smith:** Software (equal); validation (equal); writing – original draft (equal); writing – review and editing (equal). **Marshall Keyster:** Data curation (equal); resources (equal); software (equal); validation (equal); writing – original draft (equal); writing – review and editing (equal). **Ashwil Klein:** Formal analysis (equal); resources (equal); software (equal); validation (equal); writing – original draft (equal); writing – review and editing (equal).

## CONFLICT OF INTEREST STATEMENT

None declared.

## Data Availability

The data that support the findings of this study are available from the corresponding author upon reasonable request.
